# Does social isolation mediate the association between hearing loss and cognition in adults? A systematic review and meta-analysis of longitudinal studies

**DOI:** 10.3389/fpubh.2024.1347794

**Published:** 2024-01-16

**Authors:** Nisha Dhanda, Amanda Hall, James Martin

**Affiliations:** ^1^Institute of Applied Health Research, College of Medical and Dental Sciences, University of Birmingham, Birmingham, United Kingdom; ^2^Department of Audiology, College of Health and Life Sciences, Aston University, Birmingham, United Kingdom

**Keywords:** social isolation, hearing loss, cognition, mediation, meta-analysis

## Abstract

**Background:**

There has been extensive research on the relationship between hearing and cognitive impairment in older adults but little examination of the role of mediating factors. Social isolation is a potential mediator, occurring because of hearing loss, and contributing to accelerated cognitive decline. Previous systematic reviews on this topic area have not considered the temporal nature of hearing loss and cognitive impairment exclusively or examined potential mediators within a longitudinal study design.

**Methods:**

A systematic review was conducted. Electronic searches were performed in Web of Science, PubMed (Medline), Scopus, EMBASE, PsychInfo, and ProQuest (PsychArticles and ProQuest Dissertation and Theses) based on a search string of keywords relating to hearing loss, social isolation, and cognitive impairment/dementia in June 2023. Papers were critically appraised using the CASP checklists for cohort studies. Risk of bias in the selected studies was assessed using the Item Bank for Assessment of Risk of Bias and Precision for Observational Studies of Interventions or Exposures.

**Results:**

Eleven of the 15 included studies provide evidence of a dose-dependent association between hearing threshold (40 dB HL or greater) and later cognitive impairment or incident dementia. Only one study included social isolation as a mediator, which was found to not be a significant contributing factor. The meta-analysis of 5 studies pooled hazard ratio for cognitive impairment due to hearing loss is 1.11 (95% CI: 1.06 to 1.15, *p* < 0.001). The pooled hazard ratio for incident dementia due to hearing loss was HR 1.21 (95% CI: 1.11 to 1.31, *p* = 0.002).

**Conclusion:**

The analysis of included studies indicate that hearing threshold level affects later cognitive status or dementia diagnosis. There is not enough evidence to determine the role of social isolation as a mediator. Future epidemiology studies need to measure different elements of social isolation and ensure that hearing and cognition are measured at multiple time points.

## 1 Introduction

The individual consequences of age-related hearing loss extend far beyond difficulties with auditory detection (1), impacting on speech perception (2), often leading to social withdrawal, isolation, and depression because of persistently unsuccessful communication (3, 4). Associations of hearing loss with wider health outcomes have also been well documented, including cognitive decline, and dementia (5).

There is much interest in the mechanisms by which hearing loss may lead to cognitive decline (6). Key theories are the cognitive load hypothesis (7), the common cause hypothesis (8), and the cascade hypothesis (9), which includes social isolation as a mediator. According to the former hypothesis, hearing loss causes degraded auditory signals, increased cognitive resources required for auditory perceptual processing, and diversion from other cognitive tasks to effortful listening, finally ending in cognitive reserve depletion (10). Both hearing loss and cognitive decline, according to the common cause hypothesis, are the result of the same neurodegenerative process in the aging brain (4). The mechanism by which social isolation mediates the relationship between hearing loss and later cognitive impairment (11) is proposed to be due to a reduction in auditory input, impacting social interactions, thereby reducing stimulation in the cognitive centers of the brain (6), leading to cognitive decline. What’s more, unaddressed hearing loss in mid-life has been identified as the single biggest modifier of dementia risk in later-life, with social isolation also included as a modifier (12). Thus, demonstrating the interweaved and complex connections between these conditions.

Although there is some empirical support for social isolation as a mediator (i.e., an intermediate variable) between hearing loss and cognitive decline from cross-sectional population data (13, 14) there are methodological challenges to identifying whether social isolation has a role in the causal pathway. Firstly, social isolation as a concept has not been consistently defined in the epidemiological literature, and tools that have been used may not adequately measure the concept of interest. For example, some studies measure the size of a social network, but an individual may perceive their social relationships to be inadequate even though they have a sizeable social network (15). Secondly, there is the issue of reverse causation and differentiating between cognitive decline as the cause of social isolation, and social isolation as the cause of cognitive decline. Within a cross-sectional study design, it is not possible to differentiate whether cognitive impairment precedes social isolation, instead of vice versa (16), and it is unethical to use “social isolation” as an exposure variable in a randomized controlled trial. A longitudinal study design offers the most robust method to assess the role of social isolation within the causal pathway.

The systematic reviews and meta-analyses published on this topic area provide evidence to support a causal pathway for hearing loss-cognitive decline (17–19). However, many reviews include cross-sectional study designs (18), or include self-report measures of hearing (17), and so are at high risk of bias. Where longitudinal studies have been included, there is not explicit information about an adequate length of follow-up between measures. Furthermore, the role of social isolation within the causal pathway has not been investigated.

There is a need for a systematic review of prospective longitudinal observational studies with a focus on studies investigating mediating factors. This will allow mechanisms and mediators of hearing threshold and later cognitive impairment/dementia to be identified, specifically the role of social isolation.

These gaps in knowledge led to the formation of the following research questions:

1. Does hearing loss cause later cognitive impairment and/or dementia diagnosis in adults?
2. Is social isolation a mediating factor in the relationship between hearing loss and later cognitive impairment/dementia diagnosis?

**Figure 1:**
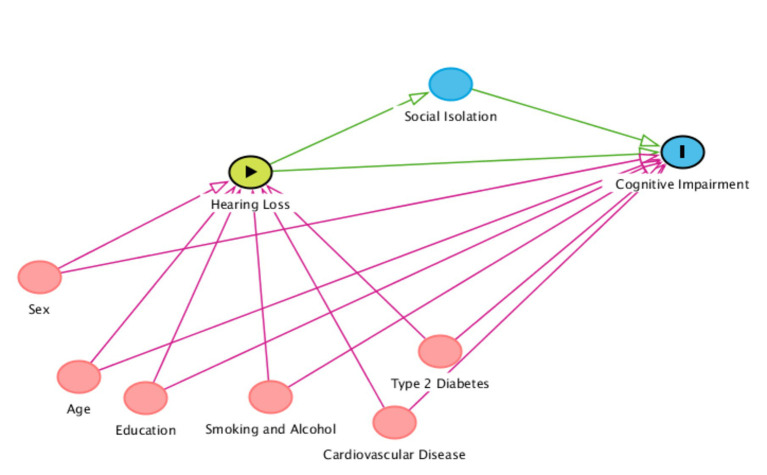
Direct acyclic graph outlining the proposed relationship between hearing loss as an exposure, social isolation as a mediator, cognitive impairment as an outcome, and potential confounders. The confounders are not an exhaustive list or limited to those outlined below. Red variables = possible confounders in chronological order. Blue variables = outcomes/mediators. Green variable = exposure. The confounders are also applicable to social isolation as an outcome.

Figure 1 shows the potential causal pathway between hearing threshold and cognitive impairment, and the hypothesized role of social isolation along the pathway. Potential confounding variables are included to demonstrate the range of factors that can influence the hearing-cognition association, and which should be accounted for in epidemiology studies.

## 2 Methods

A systematic literature search was conducted in January 2019, and an updated search was conducted in June 2023. The review has been reported according to the PRISMA checklist (20) and Conducting Systematic Reviews and Meta-Analyses of Observational Studies of Etiology (COSMOS-E) guidance on conducting systematic reviews and meta-analyses on observational studies of etiology as provided by Dekkers et al. (21). The use of COSMOS-E influenced the searching approach in the following ways: applying an iterative approach to the research question and scoping existing literature before deciding on a focused question; ensuring a variety of medical databases were searched for thoroughness; extending searching beyond electronic databases such as reference lists of relevant articles; and meticulously considering the role of confounding, selection bias, and information bias in the chosen studies.

Pre-searches to identify relevant search terms and MeSH headings related to hearing and cognition were carried out prior to the main search. Moreover, the international prospective register of systematic reviews (PROSPERO) and Cochrane library were both searched using the terms “hearing and cognition” to ensure there had been no previous systematic reviews conducted that had addressed our research questions.

### 2.1 Databases and search string

The following databases were used: Web of Science, PubMed (Medline), Scopus, EMBASE, PsychInfo, and ProQuest (PsychArticles and ProQuest Dissertation and Theses).

The following search string was used:

(hearing OR hearing-loss OR hearing-impair* OR deaf* OR sensorineural-hearing-loss OR SNHL OR presbycusis OR hearing-disorder OR age-related-hearing-loss OR inner-ear-loss OR hearing-ability OR auditory-threshold OR sensory OR audiometry) AND (cognition OR cognitive-decline OR cognitive-deficit OR mild-cognitive-impairment OR dementia OR cognitive-impairment OR cognitive-difficulty OR cognitive-defect OR Alzheimer’s-disease OR cognitive-function OR demented OR incident-dementia).

All search terms were searched in the fields for “title” or “title/abstract/keywords” as an alternative. The main search string was replicated in all databases. OpenGrey, a grey literature database, was also searched using the terms “hearing and cognition.” No filters, time, or language limitations were applied. All returned searchers were exported into Endnote X7 software where duplicates were removed. Titles and abstracts were then exported into a Microsoft Excel spreadsheet for study selection.

Eligibility criteria.

The inclusion criteria were as follows:

- Longitudinal repeated-measures studies of at least two time points to allow the temporal nature of hearing to be addressed.
- Hearing threshold measured via pure tone audiometry at time point 1 (minimum) to reduce bias from self-reported hearing.
- Measure of cognitive function at time point 1 or 2 and subsequent time points, or dementia diagnosis at subsequent time points for time of exposure and outcome.
- Adult human participants aged 18 or over.

The exclusion criteria were as follows:

- Studies using self-reported hearing loss (i.e., people identifying whether they have hearing issues with or without formal testing).
- Studies using speech threshold testing, as this does not provide a measure of hearing sensitivity influenced by language ability.
- Narrative reviews and commentaries, as empirical data was required for synthesis.
- Systematic reviews and meta-analyses.
- Animal studies.
- Dementia diagnosis present at baseline time point 1, to ensure causality could be determined between hearing and later dementia diagnosis.

### 2.2 Study selection

Using EndNote X7, two reviewers (N.D. and A.H.) independently screened titles and abstracts in duplicate (22) during the first search in January 2019. Using the established eligibility criteria, we independently evaluated full-text publications in duplicate. Regarding inclusion and exclusion criteria, both reviewers concurred. Discussion and evaluation of the inclusion and exclusion criteria were used to settle disagreements.

### 2.3 Data extraction and study quality

One reviewer (N.D.) extracted data independently from the included studies using a standardized electronic data form. A second reviewer (A.H.) independently checked a selection of the data related to the first search in January 2019. The data elements extracted included basic study information, participant demographics, the cognitive measurement tool used, and the dementia diagnosis measurement tool used. Included studies were critically appraised using the Critical Appraisal Skills Program (CASP) checklists for cohort studies (23). Risk of bias was assessed using the Item Bank for Assessment of Risk of Bias and Precision for Observational Studies of Interventions or Exposures (24). These tools were used to ensure that both quality assessment and risk of bias were considered for the included studies, appropriate to the study type.

### 2.4 Planned meta-analysis

Two *a priori* meta-analyses investigating the role of social isolation as a mediator on cognitive score and dementia diagnosis were planned (assuming the same cognitive test or dementia diagnosis is used), with the plan to pool hazard ratios/odds ratios as appropriate. However, because of a lack of studies using mediation analysis, we performed two meta-analyses (for cognitive score and dementia diagnosis). All studies that were not at high risk of bias (or red rating) were included. Pooled (adjusted model) hazard ratios were calculated using fixed-effects models, weighting using the inverse variance method. Statistical heterogeneity was assessed using the *I*^2^ statistic. Analysis was performed using RevMan 5 software (25).

## 3 Results

**Figure 2:**
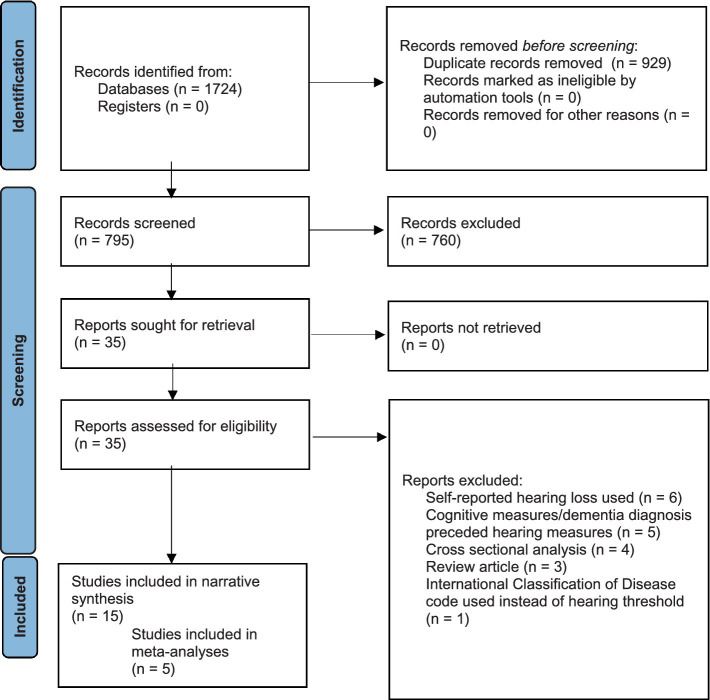
PRISMA flow diagram.

The screen and study selection process are summarized in Figure 2, which summarizes the results of the two separate searches. After duplicates were removed, 795 abstracts were screened, and after full-text review, 15 publications were eligible for inclusion. Ten studies were not included in the meta-analyses as the statistical analysis in these studies differed greatly from other studies with the same (or similar) outcome. These 10 studies are narratively synthesized.

The narrative synthesis revealed that 11 of the 15 included studies provided evidence of a dose-dependent association between hearing threshold and later cognitive impairment or incident dementia (Table 1).

**Table 1:** Summary of risk of bias using the item bank for assessment of risk of bias and precision for observational studies of interventions or exposures.

Author (year)	Are exposures assessed using valid and reliable measures?	Are outcomes assessed using valid and reliable measures?	Is the time following exposure sufficient to support the evaluation of primary outcome?	Risk of bias RAG rating Red–High Risk Amber–Medium Risk Green–Low Risk	Showed age adjusted causal relationship between HL and CD or ID Y/N/NS
Alattar et al. (2020) (26)	Yes	Yes	Yes		Y
Anstey et al. (2003) (27)	Yes	Yes	Yes		NS
Armstrong et al. (2018) (28)	Yes	Yes	No–only 2 years		Y
Croll et al. (2021) (29)	Yes	Yes	No-only 4 years		N
Deal et al. (2017) (30)	Yes	Yes	Partially–6 years		Y
Fischer et al. (2016) (31)	Yes	Yes	Yes		Y
Gallacher et al. (2012) (32)	No*	Yes	Yes		Y
Ge et al. (2021) (33)	No	Yes	Partially–max 6 year		Y
Hong et al. (2016) (34)	Yes	No–MMSE blind version used.	Yes		N
Lin et al. (2011) (35)	Yes	Yes	Partially–6 years		Y
Lin et al. (2013) (36)	Yes	Yes	Yes		Y
Lindenberger and Ghisletta (2009) (37)	Yes	Yes	Yes		Not sure
Okely et al. (2019) (38)	Yes	Yes	No–only 3 years		Y
Uchida et al. (2016) (39)	Yes	Yes	Yes		Y
Valentijn et al. (2005) (40)	Yes	Yes	Partially–6 years		Y

^*^PTA covering four frequencies and analyzed as a continuous variable but noise levels high under which hearing was tested and correlations not high/consistent (better to know degree of difference). HL, Hearing loss; CD, Cognitive decline; ID, Incident Dementia; Y, Yes; N, No; NS, yes but not significant at the 5% level.

### 3.1 Study characteristics

All included studies were prospective longitudinal studies. The studies were based in United States (46%), Europe (33%), Australia (13%) and one study was based in Japan. There were noticeable differences in the study sizes (295 to 2,336), follow-up time (2 to 24 years), follow-up frequency (1 to 6 times), and exposure and outcome definition (see Tables 2, 3).

**Table 2:** Data extraction of study characteristics for included studies.

Author (Year)	Cohort	Country	Participants including age	Total n baseline (Time-point 1)	Total n analysis
Alattar et al. (2020) (26)	Rancho Bernado study of healthy aging	United States	Wealthy community dwelling older adults living in retirement community of Rancho Bernado, San Diego. Aged 31–92 years.	1781	1,164
Anstey et al. (2003) (27)	Australian longitudinal study of aging	Australia	Sampled from South Australian electoral roll — men and those >85 years were oversampled	1,620	T2–1229 T3–457
Armstrong et al. (2018) (28)	Baltimore longitudinal study of aging (BLSA)	United States	Community-dwelling participants aged 61–98 years	319	313
Croll et al. (2021) (29)	Rotterdam Study	Netherlands	Adult residents from Ommrood area aged 58–72 years	3,590	
Deal et al. (2017) (30)	Health Aging and Body Composition (ABC) Study	United States	Community dwelling black and white older adults living in Memphis, Tennessee or Pittsburgh aged 70–79 years.	2034	1889
Fischer et al. (2016) (31)	Epidemiology of Hearing Loss Study	United States	Residents based in Beaver Dam, Wisconsin. Aged 58–74 years.	1884	1,470
Gallacher et al. (2012) (32)	Caerphilly Cohort as part of Caerphilly Prospective Study (CaPS)	Wales	Men born between 1920 and 1939 resident in neighboring towns of Caerphilly, aged 50–62 years.	1,612	1,057
Ge et al. (2021) (33)	Health and retirement study (HRS) and Aging, demographics, and memory study (ADAMS)	United States	Older adults that were part of the HRS and ADAMS cohort, aged 73–100 years.	295	268
Hong et al. (2016) (34)	Blue mountains eye study (BMES)	Australia	Suburban Australian population who was aged 49+ years resident in Blue Mountains, West Sydney	2,334	1952 at T2 1,149 at T3
Lin et al. (2011) (35)	Baltimore longitudinal study of aging (BLSA)	United States	Community-dwelling adults from and around Baltimore, USA, aged 50–84 years.	639	638
Lin et al. (2013) (36)	Health aging and body composition ABC study	United States	Community dwelling black and white older adults living in Memphis, Tennessee or Pittsburgh aged 70–79 years.	1984	1,626
Lindenberger and Ghisletta (2009) (37)	Berlin aging study	Germany	Participants living in West Berlin, aged 70–100 years.	516	46 (T6)
Okely et al. (2019) (38)	The Lothian Birth Cohort 1936	Scotland	Participants living in Edinburgh and Lothian areas of Scotland who were born in 1936, aged 76–79 years.	696	550
Uchida et al. (2016) (39)	National Institute for Longevity sciences–longitudinal study of aging	Japan	Community dwellers in Aichi Prefecture in central Japan aged 60–79 years	2,267	1,109
Valentijn et al. (2005) (40)	Maastricht aging study	Netherlands	Healthy older Dutch adults aged 55–81 years	418	391

SD, Standard deviation; T, time point.

**Table 3:** Data extraction of analysis methods for included studies.

Author (Year)	Time points	F (yr)	Exposure	Outcome	Confounders [Mediators]	Type of analysis
Alattar et al. (2020) (26)	T1 (1992–1996)– AM and CT T2-T6 every four years CT only	24	PTA 0.5–4 kHz Categorical HL	MMSE, trail-making test part, VFT at T2, T3, T4, T5, T6	LDL, HDL, lifestyle factors, physical health, depression diagnosis [social group involvement, number and frequency of contact with close friends and family, marital status]	Linear mixed-effect model
Anstey et al. (2003) (27)	T1–AM and CT T2–AM and CT, T3–AM and CT	8	PTA at 2,3 and 4 kHz Change in HL from baseline–continuous 10 dB steps	Similarities, picture naming, national adult reading test, digit symbol substitution test, symbol recall, picture recall, word recall at T2 and T3	Depression, self-rated health, physical health	Latent growth curve models using individual change scores
Armstrong et al. (2018) (28)	T1–AM and CT T2–AM and CT	2	PTA–average of 0.5–4 kHz HL continuous 10 dB steps	Trail-making test part B, digit symbol substitution test, California verbal learning test, digit span forward/backward, Benton visual retention test, MMSE at T2	Age, sex, race, vascular burden, education	Bivariate auto regressive cross-lagged models
Croll et al. (2021) (29)	T1–AM T2–CT	3–4	PTA at 0.25., 0.5, 4 and 8. HL categorical	MMSE, Stroop test, LDST at T2	Age, age squared (non-linear trend of age), sex, education, alcohol consumption, smoking status, SBP, DBP, blood pressure lowering medication	Linear mixed-effect models.
Deal et al. (2017) (30)	T1–AM T2, T3, T4–CT, DD	6	PTA–average of 0.5–4 kHz HL categorical: Normal/mild/mod/severe	Dementia diagnosis at T2, T3, and T4	Age, race, sex, education, study site, cardiovascular factors (smoking status, hypertension, and diabetes)	Cox proportional hazards models
Fischer et al. (2016) (31)	T1–AM T2, T3–CT	10	PTA–average of 0.5–4 kHz HL > 25 dB (Y/N)	MMSE at T2 and T3	Age, sex, education, smoking status, BMI, exercise, alcohol consumptions, hypertension, diabetes, number of inflammatory markers, non-HDL cholesterol, mean IMT, frailty score	Cox proportional hazards models
Gallacher et al. (2012) (32)	T1–AM T2–CT T3–AM and CT, T4–CT and DD	17	PTA–average of 0.5–4 kHz HL continuous 10 dB steps	Decline in cognitive score at T2 and T4 or dementia diagnosis at T4	Age, social class and anxiety, premorbid cognitive ability score	Logistic and Linear fixed-effect models
Ge et al. (2021) (33)	T1–AM T2, T3, T4 – CT	8	PTA at 0.5,1,2,4 kHz HL > 25 (y/n)	Telephone interview for Cognitive Status (TICS). Vision loss, dual sensory loss at T2, T3, T4	Education, race, survey wave, number of health conditions, physical exercise	Linear mixed-effects models
Hong et al. (2016) (34)	T1–AM and CT, T2–AM and CT, T3–AM and CT	10	PTA–average of 0.5–4 kHz HL > 40 dB (Y/N)	MMSE-Blind at T2 and T3	Baseline age and sex, walking disability, living arrangements, home ownership, education, baseline MMSE score, ≥3 major comorbidities, depressive symptoms	Logistic fixed-effect models
Lin et al. (2011) (35)	T1–AM T2–DD	17	PTA–average of 0.5–4 kHz (better hearing ear) HL categorical: Normal/mild/ mod/severe	Dementia diagnosis at T2	Age, sex, education, sex, age, race, education, diabetes, smoking, and hypertension. Additional models had baseline Blessed scores and hearing aid use.	Cox proportional hazards models
Lin et al. (2013) (36)	T1–AM and CT T2, T3, T4–CT	6	PTA–average of 0.5–4 kHz (better hearing ear) HL > 25 dB (Y/N)	3MS at T2, T3, T4	Age, sex, race/ethnicity, education, study site, cardiovascular risk factors (smoking status, hypertension, diabetes and stroke history) [depression]	Linear mixed-effects models
Lindenberger and Ghisletta (2009) (37)	T1–T6–AM T1, T3, T4, T5, T6–CT	13	PTA at 2,3,4 and 6 kHz (averaged)	Digit letter, identical pictures, paired associates, memory for text, category, word beginning, vocabulary, spot a word at T3, T4, T5, T6	Time to death, risk of dementia	Linear and non-linear models
Okely et al. (2019) (38)	T1–AM and CT, T2–AM and CT	3	HearCheck at 1 and 3 kHz	Spatial span, matrix reasoning, block design, symbol search, digit symbol substitution test, inspection time test, four choice reaction time test, digit span backwards, verbal paired associated, logical memory, national adult reading test, phonemic verbal fluency at T2	Age, sex, childhood cognitive ability, occupational social class, symptoms of anxiety and depression, smoking status, hearing aid use, history of diabetes, cardiovascular disease, stroke and hypertension	Latent change score model
Uchida et al. (2016) (39)	T1–AM and CT T2–CT	13	PTA–average of 0.5–4 kHz (better hearing ear) HL > 25 dB (Y/N)	Information, similarities, picture completion, digit symbol substitution at T2	Age, sex, education, medical history of hypertension, diabetes, stroke, cardiac disease, current smoking status, marital status, and occupation	Linear mixed-effects models
Valentijn et al. (2005) (40)	T1–AM and CT, T2–AM and CT	6	PTA at 1,2 and 4 kH HL continuous 1 dB steps	Visual verbal learning test, stroop, color word test, concept shifting task, VFT LDST at T2	Age, education, sex, baseline performance	Hierarchical linear regression

Demographics (age, sex, gender, education, race) were confounders in all studies. PTA, Pure Tone Average; CT, Cognitive testing; AM, Audiometry; DD, Dementia Diagnosis; T1, Timepoint 1; HL, Hearing loss; LDST, Letter-Digit Substitution Test; VFT, Verbal Fluency Test; MMSE, Mini-Mental State Examination; Lifestyle factors (smoking, alcohol, exercise), physical health (hypertension, diabetes, stroke); F, Follow up.

Of the 15 included papers, two studies exclusively used dementia diagnosis as an outcome (30, 35), 12 studies used cognitive score as an outcome (26–29, 31, 33, 34, 36–40), and one study used both (32).

### 3.2 Mediation

One study identified depression as a potential mediator (36), but only one study (26) describes using mediation analysis. Alattar et al. (26) examined possible mediation by social engagement by including interaction terms in a mixed-model framework. This study described the relationship between hearing impairment and cognitive test performance when social engagement is considered although they do not capture the proportion of the relationship between hearing impairment and cognitive test performance that is mediated by social engagement – often referred to as the indirect effect. The presence of a relationship in the adjusted model would indicate that social engagement is not a complete mediator, but the lack of reporting of the indirect effect means we cannot rule out social engagement as a partial mediator. Furthermore, one study used a potential mediator variable “lives alone” incorrectly as a confounder (34).

### 3.3 Risk of bias assessment

The Item Bank for Assessment of Risk of Bias and Precision for Observational Studies of Interventions or Exposures (24) was used for the qualitative assessment of the included studies. The analysis revealed that only 6 (40%) studies were free of any biases. The risk of bias assessment does not include a formal rating or scoring system like other assessment tools. The purpose of the tool is to consider the believability of study results across a wide range of factors. The reviewer has the discretion to interpret the levels of bias within the context of the other studies included in the review, and within the context of the topic area. A red, amber, green (RAG) rating was added to aid the reader in the overall levels of bias within each study. For example, if the duration between exposure and outcome measures were less than 10 years, then a study would have an amber rating. Similarly, if there are not valid and reliable measures used for the exposure or outcome, then an amber rating would be given. If there were four or more occurrences to warrant an amber rating, then a study would have a red rating. This did not occur in any of the included studies. Therefore, there was a combination of low bias (green rating) and medium bias (amber rating) studies included within the review. Similar risk of bias assessment tools, such as Risk Of Bias In Non-randomized Studies - of Exposure (ROBINS-E) use formal RAG rating software to assist with the interpretation of high, medium, and low risk of bias studies included within the review. For example, ‘robvis’ software was produced by McGuinness and Higgins (41) for this purpose. However, on balance, the Item Bank for Assessment of Risk of Bias and Precision for Observational Studies of Interventions or Exposures tool was the most appropriate for the included cohort studies.

None of the studies had a high risk of bias but 60% of the studies were at a moderate risk of bias due to reporting bias, information bias, selection bias, attrition bias, or diagnostic bias. The detailed assessment sheet is provided in the appendix with author’s comments and analysis. Most studies did not report the inclusion and exclusion criteria clearly (26–28, 34), some studies also did not provide the detailed account of exposure measurements (37, 40). Only three studies addressed the attrition rate using sensitivity analysis (27, 31, 37) and three studies did not have a high attrition rate (26, 36, 39).

### 3.4 Hearing ascertainment

Pure tone audiometry (conventional or screening method) was the method of obtaining hearing levels in all included studies. However, there was variation in the definition of hearing loss, and whether it was explicitly defined in the methods. Most studies measured hearing at baseline only, while some measured hearing at different time points and used the change in hearing as a predictor. Hong et al. (34) defined hearing loss as the pure-tone average of 0.5, 1, 2 and 4 kHz being greater than 40 dB HL, while Fischer et al. (31), Lin et al. (36), and Uchida et al. (39) defined hearing loss as the pure-tone average of 0.5, 1, 2 and 4 kHz being greater than 25 dB HL. Alattar et al. (26), Deal et al. (30), and Lin et al. (35) all defined hearing loss in categorical terms where normal hearing was less than 25 dB HL, mild as 25-40 dB HL, moderate as 41-70 dB HL and severe as greater than 70 dB HL for the pure-tone average of 0.5-4 kHz in the better ear. Deal et al. (30) and Alattar et al. (26) combined moderate–severe hearing loss as greater than 40 dB HL. Lin et al. (35) also used hearing threshold as a continuous variable, as did Gallacher et al. (32) and Armstrong et al. (28) who used 10 dB steps but did not define hearing loss in their methods, while Valentijn et al. (40) used 1 dB steps also without a definition of hearing loss.

Hearing was measured at three time-points in Anstey et al. (27) and Hong et al. (34), two time-points in Gallacher et al. (32), and one time-point in all other studies. A change in hearing was measured by Gallacher et al. (32) and Anstey et al. (27), but not by Hong et al. (34).

Okely et al. (38) used a hearing screening device at 40 dB HL instead of conventional pure tone audiometry, with hearing then categorized based on number of tones (out of six) that are heard.

These differences in how hearing loss has been defined provide an increased risk of misclassification bias within the selected studies and can make comparing and generalizing findings difficult.

### 3.5 Dementia ascertainment

Deal et al. (30) defined incident dementia as the use of a prescribed dementia medication, identification of diagnosis from hospital records, or a race-stratified Modified Mini-Mental State Examination (3MS) score decline more than 1.5 standard deviations from the baseline mean. Lin et al. (35) defined dementia using the Diagnostic and Statistical Manual of Mental Disorders (DSM) (Third Edition Revised) and National Institute of Neurological and Communicative Disorders and Stroke-Alzheimer Disease and Related Disorders Association (NINCDS-ADRDA), criteria for diagnosing Alzheimer’s disease. While Gallacher et al. (32) also used NINCDS-ADRDA and DSM (Fourth Edition), in addition to National Institute of Neurological and Communicative Disorders and Stroke-Association Internationale pour la Recherche et l’Enseignement en Neurosciences (NINCDS-AIREN) criteria for vascular dementia diagnosis.

### 3.6 Cognitive tests

When cognitive test score was the primary outcome, the most frequent cognitive test used was the Mini-Mental State Examination (MMSE) with a cut-off score of 24 out of 30 for cognitive impairment (5 of 10 studies), MMSE is primarily used as a screening tool within clinical practice and is often criticized for not being specific enough to detect lower levels of cognitive domains associated with various dementias (42). Hence, the use of MMSE as a cognitive test was questionable. Having said that, as a relatively quick and easy tool to administer, it is used to assess a broad range of cognitive domains. Variations of MMSE included 3MS (a longer version of MMSE with a broader range of scoring from 0 to 100), and MMSE-Blind where visual elements were taken out. After MMSE, the Digit Symbol Substitution Test, a processing speed test, was often included in the battery of cognitive tests. Since problems with recall and processing speed are often initial symptoms of dementia, these tests may be well suited to the detection of cognitive decline (43). Tests of immediate and delayed recall were used by Gallacher et al. (32) and Anstey et al. (27), and Trail Making Test Part B (used to assess executive function) was used in Valentijn et al. (40), Alattar et al. (26), and Armstrong et al. (28). Okely et al. (38) used the greatest number of cognitive measures in their study, most of which were subsets of the Wechsler Adult Intelligence Scale tests.

A variety of cognitive tests were used in the included studies. Some tests (or components of tests) were administered verbally. This could have biased participants with hearing impairments, who answered questions incorrectly from not hearing rather than not knowing. Specifically, components of the MMSE, tests of immediate and delayed recall (Rivermead Memory Scales), and California Verbal Learning Test may have affected participants’ performance. Some studies reported that those administering the tests had appropriate training in communication techniques (i.e., ensuring to face the participant when speaking in a well-lit environment). However, it is difficult to conclude whether this is enough to prevent those with hearing loss from being disadvantaged when undergoing cognitive assessment. Furthermore, it is unclear whether participants could use hearing aids while undergoing cognitive assessment. If this occurred, it would present a higher risk of bias.

### 3.7 Attrition rates

Overall, the attrition rates in the included cohorts were lower than 30%, with the main reasons for the missing data being that participants did not attend due to death (26), relocation, cognitive tests not completed at follow-up (36), or hearing corrected by use of a hearing aid (34), thereby making a person ineligible to continue to participate. However, only two studies (27, 32) provided information on the characteristics of participants who did not receive follow-up. Therefore, it is unclear whether those participants who were not followed through to the final timepoint in the other studies, had dropped out due to poorer health and disease burden or volunteered to do so for another reason. Usually, the attrition rates are dealt with through sensitivity analysis or full-information maximum likelihood-based statistical methods, as done by Anstey et al. (27). An inability to address high attrition rates in any statistical analysis will increase the risk of attrition selection bias within studies, leading to findings that lack external validity and incorporate collider bias, i.e., when the selection of study participants or the way data is analyzed introduces bias by conditioning on a common effect of two or more variables.

### 3.8 Selection bias

All studies provide the inclusion and exclusion criteria of the original cohorts from which participants were selected and the sub-cohorts used for the analysis. Yet, initial recruitment of those cohorts may not be entirely representative of the older adults within the countries where the studies were conducted regarding race, gender, and age. For example, Lin et al. (36) used the Health ABC Study for their analysis, recruiting only participants of white and black ethnicity. Including more ethnicities within the study may have influenced the results (introducing collider bias), as a greater proportion of participants would be exposed to the included confounders, leading to incorrect results.

### 3.9 Choice of longitudinal cohort

Deal et al. (30) and Lin et al. (36) both used the Health ABC Study of Aging cohort dataset, but they used different primary outcomes: dementia diagnosis versus cognitive decline, respectively. Although Deal et al. (30) included analysis of cognitive test scores, these scores were conducted earlier than the audiometry measures, so did not meet the inclusion criteria of the review. Similarly, the same cohort dataset (Baltimore Longitudinal Study of Aging) was used by Lin et al. (35) and Armstrong et al. (28) but Lin et al. (35) used incident dementia as the primary outcome, whereas Armstrong et al. (28) used change in cognitive score. More than double the number of participants were used in the analysis carried out by Lin et al. (35), as compared to Armstrong et al. (28): 638 versus 313, respectively. This increase in number of participants was largely to the difference in follow-up time periods used in each analysis (11 years vs. 2 years) and the number of participants who had undergone all cognitive tests during the 2012–2017 period of data collection that Armstrong et al. (28) was based on.

### 3.10 Confounding variables

All the included studies used some or most of the confounders identified in the proposed DAG (Figure 1). The main confounding variables used in the included studies were age, sex, race/ethnicity, education, hypertension, diabetes, stroke history, and smoking status. Other studies included confounders such as depressive symptoms, alcohol consumption, occupation, marital status, frequency of contact with close family and friends, and social group involvement. Using variables such as depression and social group contact and involvement is not appropriate within the context of a hearing-cognition causal pathway since they do not meet the definition of confounders, i.e., directly influencing both the exposure and outcome but not on the causal pathway. There is no evidence to suggest that depression or social group contact/involvement causes hearing loss. Depression and social group involvement were also used as mediators by Lin et al. (36) and Alattar et al. (26), respectively although they only considered these mediators at one point in time and did not consider how they could change over time.

In some of the included studies (30, 35, 36), separate analyses were conducted for participants using hearing aids. They did not find reduced risk of dementia or cognitive decline with hearing aid use. Although the estimations were in the anticipated direction of reduced risk, they had wide confidence intervals and did not achieve statistical significance, due to small sample sizes.

One study used a potential mediator variable “lives alone” as a confounder in their statistical analysis (34). Living alone can be used as a proxy measure for loneliness/social isolation, which may mediate the hearing-cognition relationship. Therefore, using the variable ‘lives alone’ as a confounder within the model, could provide an inaccurate estimate and interpretation of the strength of the hearing-cognition relationship.

### 3.11 Statistical analysis methods

The association between hearing impairment and cognition were mostly evaluated using mixed-effects regression models. No studies accounted for missing outcome or covariate data.

Alattar et al. (26) used change scores of cognitive tests as an average measure between two time points.

### 3.12 Meta-analysis

**Figure 3:**
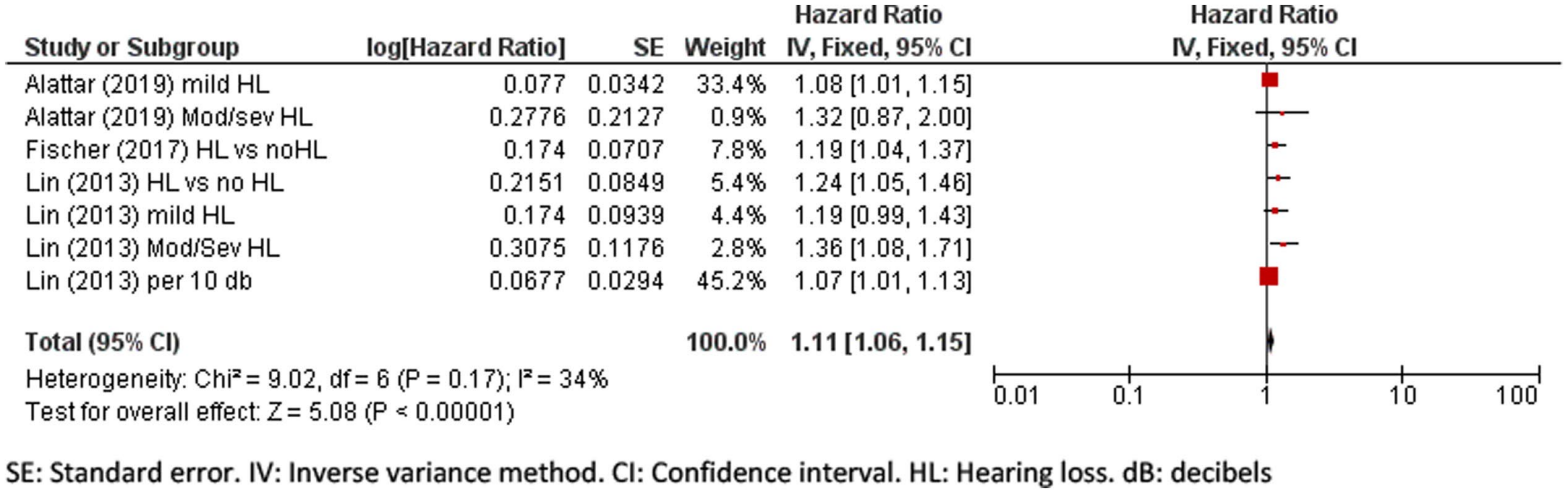
Forest plot showing the impact of hearing loss on cognitive impairment. SE, Standard error; IV, Inverse variance method; CI, Confidence interval; HL, Hearing loss; dB, decibels.

A meta-analysis of five eligible studies was performed to pool the effect of hearing loss on cognitive impairment and dementia incidence. The pooled hazard ratio for cognitive impairment due to hearing loss is 1.11 (95% CI: 1.06 to 1.15, *p* < 0.001; Figure 3) indicating that people with hearing loss have an increased hazard of 11% of developing cognitive impairment.

**Figure 4:**
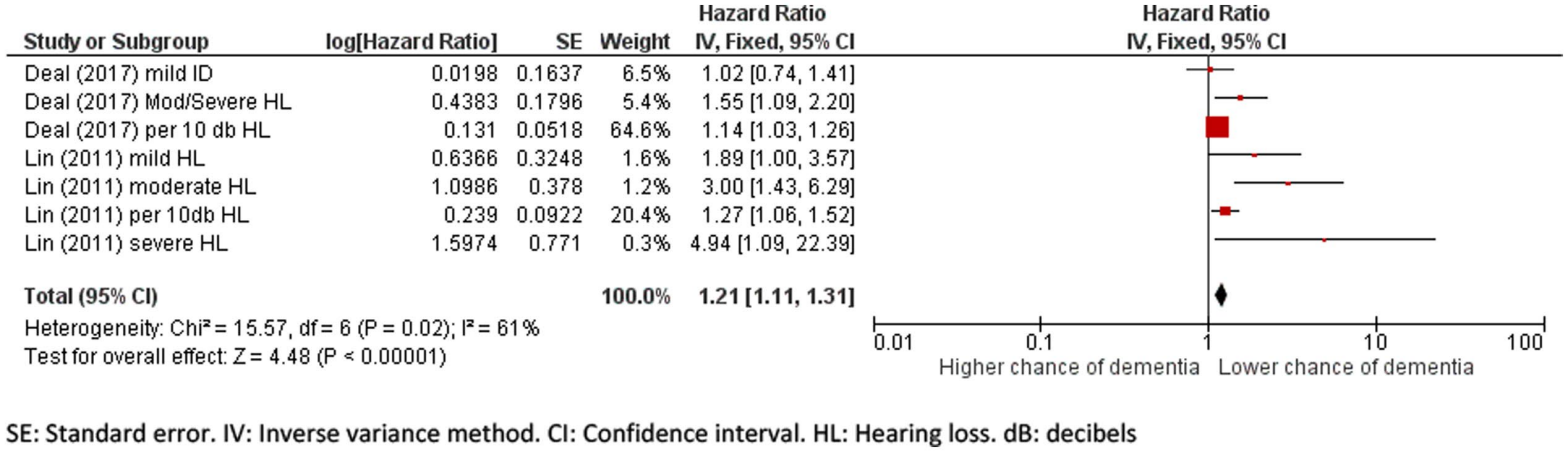
Forest plot showing the impact of hearing loss on incidence of dementia. SE, Standard error; IV, Inverse variance method; CI, Confidence interval; HL, Hearing loss; dB, decibels.

The pooled hazard ratio for incident dementia due to hearing loss was 1.21 (95% CI: 1.11 to 1.31, *p* = 0.002; Figure 4) and there was a high percentage of total variability due to between-study heterogeneity (*I*^2^ = 61%). The differences in the severity of hearing loss across groups might have added to the heterogeneity, limiting the validity of results.

## 4 Discussion

### 4.1 Main findings

This is the first systematic review to include only longitudinal studies of hearing and cognitive status and dementia and investigate social isolation as a mediator. The analysis of included studies indicate that level of hearing threshold affects later cognitive status or dementia diagnosis. A causal relationship between hearing loss and incidence of dementia and cognitive impairment was found in 11 out of 15 studies. The pooled hazard ratio also confirmed that hearing loss has a statistically significant impact on cognitive decline (HR 1.11), of which the clinical importance is debateable. The pooled hazard ratio for hearing loss and incident dementia was slightly higher at 1.21. These findings answer the first research question proposed in this study. But how and why hearing loss and cognitive impairment are associated with each other, can only be explained through mediation analysis.

The second research question of this systematic review was to identify the studies investigating mediation of hearing loss and cognitive impairment through social isolation. Only one study (26) was identified that fit the inclusion criteria, but unfortunately it had not fully reported the results of a mediation analysis. Alattar et al. (26) determined social engagement or isolation levels using the frequency of contacts with friends/family, and number of close friends/family, which is an unvalidated and crude measure. Incidentally, the authors found no significant differences in social engagement measures between those with and without hearing impairment, and inclusion of the social engagement measures within the statistical models did not weaken any of the observed associations. This suggests that hearing-impaired individuals who remain socially active may experience accelerated cognitive decline. Research has been conducted using self-reported hearing loss (44) to determine whether social isolation links hearing loss with cognitive decline and has supported social isolation as a mediator. After adjusting for several psychosocial factors, such as depression, social network, and psychotropic consumption, they found that cognitive decline in individuals with hearing impairment was no longer significantly different. This implies that hearing loss does not have a direct effect on cognitive decline, but that depressive symptoms and social isolation mediate this association. Therefore, hearing aids may help improve mood, increase social interaction, and enable participation in cognitively stimulating activities, which could potentially slow cognitive decline. Of note, is the different measures of social isolation used by Alattar et al. (26) and Amieva et al. (44), evidencing an increasing need for standardized and applicable social isolation measured in longitudinal studies. Furthermore, the use of self-reported hearing assessment places the study at a high risk of bias. Self-reported hearing measures also add subjectivity to the study and increase inaccurate measures that underestimate associations with other variables (45). As the inclusion criterion was standardized PTA testing to ascertain hearing loss, Amieva et al. (44) was excluded at screening stage. Having said that a recent study has shown the importance of using subjective hearing questionnaires together with hearing tests to provide a better understanding of the hearing difficulties of older adults with cognitive impairment (46). They found self-report questionnaires scores among cognitively impaired older adults may predict their hearing difficulties. However, self-report questionnaires assessing peripheral hearing difficulties using PTA may not be valid in this group since the correlation of the questionnaire results with the objective hearing test is weaker than in those with normal cognition. This was not an issue for our investigation, as we were interested in studies measuring hearing thresholds prior to the detection of any cognitive impairment.

Mediation analysis is an analytical approach which could help determine whether an observed relationship between hearing and cognition can be explained through social isolation. In this context, studies should look to establish what relationships exist between hearing, social isolation, and cognition. It is recommended that studies conducting mediation analysis report direct effects (the impact of hearing on cognition) accounting for social isolation, and the indirect effect (the proportion of the relationship between hearing and cognition that is mediated by social isolation), which will help inform whether social isolation acts as a mediator or not in the relationship between hearing and cognition. There are several papers that cover the methods required for mediation analysis (47–49).

### 4.2 Validation of findings

The findings on the association of hearing loss and dementia or cognitive impairment of our systematic review aligns with the findings of other similar studies, which also depicted an association between hearing impairment and the incidence of dementia (17, 18, 50). The review by Ford et al. (50) demonstrated a hazard ratio of 1.49 (95% CI 1.30–1.67) on dementia for those with hearing impairment. This was higher than the pooled effect reported here but still followed the same direction. They included 14 studies in their meta-analysis, one of which was their own prospective cohort study of almost 40,000 older men. The review by Loughrey et al. (18) of cross-sectional and longitudinal studies reported odds ratios (comparing hearing loss with dementia and cognitive impairment) similar in magnitude to the hazard ratios presented here. However, the results by Loughrey et al. (18) were much more uncertain, possibly because of including cross-sectional studies within the review. A larger magnitude of effect (HR = 1.59) between hearing loss and dementia was found by Liang et al. (17). While this review contained a greater number of studies, some used self-report hearing loss instead of pure tone audiometry, which may have biased the results.

A recent systematic review and meta-analysis investigating the association of age-related hearing loss with cognitive decline and dementia in English and Chinese speaking populations also reported similar results (51). This study was specifically interested in populations who spoke Sinitic-tonal languages, to see whether the hearing-cognition causal inference was supported. The authors included both objective and subjective hearing assessment in their inclusion criteria. They found that the odds of cognitive decline and dementia increase with hearing loss by 1.85 and 1.89 times through an analysis of 25 studies, but the speaking language was not a factor (51). Similar conclusions have been made by previous systematic reviews and meta-analyses even though they have not been rigorous in their inclusion and exclusion criteria, as cross-sectional data and self-reported hearing loss studies have been included (18, 19, 52).

The meta-analysis was performed on a minimal number of studies thus, the results may be difficult to generalize. A meta-analysis of all the studies was not possible due to differences in the measurement of cognitive status, differences in defining and categorizing hearing loss, and statistical methods to calculate associations. It is of note that the quality of the included studies was high or medium, and the exposure variables were measured appropriately using a variation of pure tone audiometry.

There is a need to use more standardized methods and analyses to study the effect of hearing loss on dementia incidence and cognitive decline in longitudinal studies so that a pooled effect can be measured. It should also be noted that the studies that did not show a significant effect of hearing loss for example Hong et al. (34) could not be included in the meta-analysis as they reported their findings in odds ratio rather than hazard ratio and did not provide enough information to calculate a hazard ratio.

Overall, the studies used appropriate methods for assessing the impact of hearing loss on cognitive decline and vice versa but the lack of standardized outcome measures, different follow up times, high attrition rates, use of different statistical analysis make them difficult to compare. A narrative synthesis of individual studies indicated that 11 studies showed a significant association of hearing loss and cognitive decline. These studies also provided evidence that hearing loss precedes cognitive decline and may be a modifiable solution for preventing cognitive decline. The analysis of the risk of bias indicates that the studies need to make their reporting much more explicit and transparent.

### 4.3 Social isolation as a mediator

The literature regarding mediation of social isolation in hearing loss and cognition studies is very sparse despite social isolation being largely evidenced as a negative health outcome of hearing loss (11). Evidence for associations between social isolation and cognitive impairment are also widespread (53). Thus, there is a need for longitudinal studies to investigate the mediating role of social isolation on cognitive impairment and hearing loss. In future studies, social isolation should be measured at several timepoints to allow for mixed-effects longitudinal analyses, and mediation analysis if the timepoints are appropriate.

Similarly, cross-sectional data has been used as evidence of the presence of mediation (13, 14), but mediating factors are usually revealed temporally (48) thus a sequential assessment through longitudinal studies can increase the reliability of the mediation effect (54). They can also generate biased results as demonstrated by O’laughlin et al. (54) Mackinnon and Luecken (55), and Maxwell et al. (56) through careful analysis of previous studies, where they concluded that cross-sectional studies can over-estimate the mediation of a variable or produce a false-positive mediation effect. Instead, longitudinal mediation models such as cross-lagged panel and latent difference score models are suggested to identify complete or partial mediation of a variable (54, 56).

Several studies (13, 57) investigating the association between social isolation, hearing, and cognition were excluded from our analysis as they used self-reported hearing loss that can be inaccurate. Maharani et al. (57) depicted the mediating role of social isolation and loneliness between hearing loss and episodic memory scores, but they used self-reporting hearing measures which as described previously is not accurate (13). used structural equation modeling in cross-sectional data of the United Kingdom Biobank to determine whether hearing aid use, social isolation, and depressive symptoms were mediators in the association between hearing loss and cognition. Their findings suggested a positive effect of hearing aid use on cognition, but this effect was not associated with reducing social isolation or depressive symptoms albeit investigated using cross-sectional methods. Brewster et al. (58) conducted a pilot randomized controlled trial investigating whether hearing aids improve mood and cognition. Their reason for including mood as an outcome was to unpack the potential mechanism and role of depression as a mediator between hearing loss and dementia. Hearing aids were not shown to influence social isolation and depression. This could be because hearing aid use can promote social withdrawal, due to excessive amplification of background noise in social situations or may be due to inappropriate measures of social isolation.

### 4.4 Role of hearing aids

A prior longitudinal investigation indicated that hearing aids mitigated the impact of hearing loss on cognitive deterioration (44). Findings from the recent Aging and Cognitive Health Evaluation in Elders (ACHIEVE) (59) trial included two unique study populations: people who had previously participated in a heart health study and healthy volunteers who were recruited from the community. The participants in the heart health cohort benefited the most from the hearing intervention. These participants were older and had a higher risk of cognitive deterioration. Over a three-year period, the hearing intervention lowered cognitive change by 48% compared with the health education control group [difference 0·191 (0·022 to 0·360); *p* = 0·027]. The hearing intervention had no effect in reducing cognitive decline in the newly recruited healthy volunteer group after 3 years, most probably because cognitive decline based on thinking and memory is much slower in healthy aging individuals. The authors did not consider mediation and did not measure social isolation to examine the exposure-outcome effects as part of the study. Randomized controlled trials of hearing aids for cognitive decline should include evaluation of mediating factors, especially social isolation since this is the mechanism by which the hearing aids are most likely to support and positively influence (13).

A recent meta-analysis found that hearing aid users had lower levels of cognitive decline than those with unmanaged hearing loss (60). They reported a hazard ratio of 0.81 (95% CI 0.76, 0.87), indicating lower risk of decline for hearing aid user participants. While this appears encouraging, results must be interpreted with caution. Hearing aids are not a “one size fits all” solution for older adults with hearing impairment. The additional work and burden of managing hearing devices, processing sound through them, and the overall listening effort may not be of value to some individuals (61). Thus, a holistic approach to hearing healthcare would better support older adults (62) and randomized controlled trials of hearing aids to limit cognitive decline should include measures of potential mediators to examine the causal processes.

Hearing aid wearers with Alzheimer’s disease have not shown enhanced cognitive performance in prior randomized controlled studies (63), but further studies like ACHIEVE will help to clarify this position. Use of hearing aids has shown a delay in dementia incidence (64, 65) thus, monitoring hearing threshold regularly after 50 years of age can help prevent or delay dementia and cognitive impairment, which is recommended by World Health Organization (66). They have estimated the return on investment from hearing screening for adults aged above 50 years and indicated that in a high-income setting, every dollar invested in hearing screening among older adults could yield a possible return of 1.62 International dollars. Many older adults living with dementia will have hearing loss, regardless of the role that hearing aids play in the prevention of dementia or the underlying mechanisms that link hearing loss and dementia (67). Therefore, there is an urgent need for research into treatments that will improve the health of those who have dementia and hearing loss as well as their carers (12). Additionally, a recent analysis of United Kingdom Biobank cohort data has shown that those wearing hearing aids have a similar risk of dementia as in people without hearing loss (68). They analyzed the role of self-reported social isolation, loneliness and mood and found 1·52% of the total association between hearing aid use and dementia was mediated by improving social isolation, 2·28% by improving loneliness, and 7·14% by improving depressed mood. With the hypothesis that good hearing loss care could prevent up to 8% of dementia cases, their findings suggest a necessity to address hearing loss to improve cognitive decline, while acknowledging the role of mediators in the pathway.

### 4.5 Reverse causality

Despite the above, reverse causality should not be ruled out. There is some evidence of cognitive decline leading to peripheral hearing decline. In a study to determine the predictors of longitudinal hearing decline in older adults, Kiely et al. (69) found an association between the presence of cognitive impairment and faster rates of decline in peripheral hearing. The MMSE was used to measure cognitive impairment in most cohort studies that include cognitive testing, as it provides a measure of global cognitive function. However, further research is required to investigate the specific areas of cognitive function responsible for hearing decline, or because of hearing decline in older adults. Genetic data from United Kingdom Biobank (70) has been analyzed to investigate whether cognitive ability predicts hearing loss. Over 80,000 participants aged 55 and older had undertaken a measure of speech-in-noise that allowed a speech reception threshold (SRT) to be calculated. A genetic risk score for Alzheimer’s disease was also calculated and used to predict SRT. An odds ratio of 1.06 (95% CI 1.01,1.11) was calculated, which demonstrated a statistically significant association between higher Alzheimer’s Disease genetic risk score and poor speech-in-noise hearing. Therefore, a shared biological mechanism via neurodegeneration may be responsible for this finding, but genetic predictors of hearing loss must also be applied in future research to determine the true direction of causality.

### 4.6 Recommendations

There remains a need for further epidemiological analysis to be conducted where hearing threshold data is available longitudinally at several time points, and later cognitive testing or dementia screening and diagnosis of at least 10 years follow-up. Only one study, (32), measured dementia and cognitive decline at all time-points. To appropriately assess for mediation, social isolation variables must be determined at a time point in between initial hearing testing and later cognitive testing and dementia incidence. These variables should explicitly capture the concepts of social isolation beyond the simplicities of a person living alone or their marital status. Having said that, finding such a dataset may prove very difficult. While hearing, dementia, and cognitive tests are common measures in large-scale cohort studies, social isolation measures are less common. Where social isolation measures exist in cohort studies, they may be measured inaccurately at time-points between hearing and cognition, for mediation to be conducted. If this is not possible, then it may be of value to separately assess the relationship between hearing threshold and later social isolation and hearing threshold and cognitive score. This would provide supporting evidence to determine the individual relationships, which can be compared to the included studies within the review, and for randomized controlled trials investigating hearing aid use and cognition.

What’s more, exploratory work related to the lived experience of social isolation in older adults would help to determine the appropriate mediating variables to understand the mechanisms underlying hearing threshold and later cognitive impairment. When considering intervention development, those that are only hearing aid focused may not see an effect if social isolation is the mediator. Thus, highlighting the need for comprehensive exploratory research to be conducted.

## Data availability statement

Publicly available datasets were analyzed in this study. This data can be found here: The data analyzed for this study have been derived from publicly available research journal articles.

## Author contributions

ND: Conceptualization, Formal analysis, Methodology, Writing – original draft. AH: Conceptualization, Formal analysis, Methodology, Supervision, Writing – review & editing. JM: Formal analysis, Methodology, Writing – review & editing.
